# Rethinking Strategies for a Pharmaceutical Approach to Pain Related to Connective Tissue–Related Raynaud Phenomenon in the United States

**DOI:** 10.1002/acr.25660

**Published:** 2025-12-12

**Authors:** Tracy M. Frech, Charles G. Frech, W. David Merryman, Andrew Sternlicht, Justin Baba

**Affiliations:** ^1^ Vanderbilt University Medical Center and Tennessee Valley Healthcare System Nashville Tennessee; ^2^ Vanderbilt University Nashville Tennessee; ^3^ Tufts University School of Medicine Boston Massachusetts

## Abstract

**Objective:**

There are no US Food and Drug Administration–approved therapies for Raynaud phenomenon (RP) in the United States. Clinical trials have been challenged by study design. Important advances in RP patient‐reported outcome measures and mechanistic quantification allow RP‐related pain characterization. The rationale for this narrative review is current RP treatment guidelines that focus on vasodilation.

**Methods:**

The question of why there are limitations to RP treatment in the United States is addressed through a comprehensive search strategy of published RP treatment guidelines up until September 1, 2025. Search databases included Medline (PubMed), Embase, and Scopus for the index terms “Raynaud's phenomenon treatment guidelines.” If a society guideline was updated, only the most recent was included. Eligibility, data extraction, risk of bias, and quality assessment were subject to review by two independent reviewers, with a third reviewer resolving discrepancies. US‐specific considerations of published guidelines are reviewed.

**Results:**

A total of 118 published articles were identified by the search terms “Raynaud's phenomenon treatment guidelines,” and 27 abstracts were reviewed. Four articles published as RP treatment recommendations or guidelines were reviewed for full content. Pain management for RP is not included in guideline‐based care.

**Conclusion:**

There are advances in outcome measures for quantifying pain now available for RP clinical trials. Large US‐based registries for systemic sclerosis using patient‐reported outcomes can allow serial data collection on RP and RP‐related digital lesions to provide real‐world data on medication efficacy for pain relief.

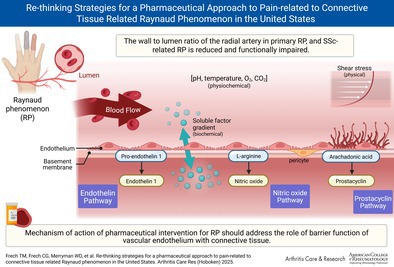

## Graphical abstract

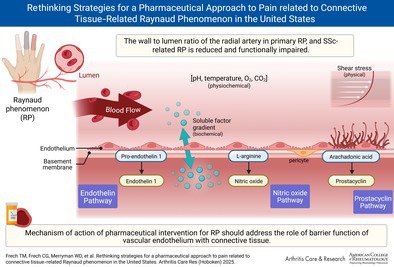

## Introduction

Raynaud phenomenon (RP) is a common painful neurosensory symptom related to digital (finger and toes) vasospasm in response to cold temperature and emotional stress, and it has a prevalence of approximately 8% in the United States.^1^ RP is a symptom complex related to digital vascular compromise, which always includes blanching triggered by cold exposure but is associated with various conditions with different outcomes.^2^ Primary RP, although symptomatically bothersome, is not associated with vascular damage or autoantibodies,^3^ whereas secondary RP, related to a connective tissue disease (CTD), typically exhibits abnormal nailfold microscopy and in its severe form, most commonly related to systemic sclerosis (SSc), results in vasculopathy‐driven digital lesions. RP is often the earliest symptom of autoimmune disease and is diagnosed by performing a physical examination of the hand, specifically by assessing digital pitting, ulceration, and telangiectasias and performing nailfold capillaroscopy. History and physical examinations can be helpful in distinguishing painful mimics of RP. Ancillary studies such as hand radiographs can identify severe autoimmune disease features such as digital tip resorption (acro‐osteolysis) and calcinosis, which are thought to be a tissue response to sustained or prolonged hypoxia and possibly prevented with vasodilator therapy.^4^ If results of the physical examination or ancillary studies are consistent with RP and abnormal, laboratory testing can identify CTD autoantibodies. In fact, a patient with RP, digital lesions (pits and/or ulcers), abnormal capillaroscopy, and an SSc autoantibody meets classification criteria for this CTD, which has among the highest morbidity and mortality of all rheumatic diseases.^5^ Thus, it is critically important to standardize the screening and treatment of RP, specifically if treatments are preventive.^6^ Ethnic, cultural, and clinical phenotypic factors appear to contribute to geographic variation in RP symptom burden in SSc, highlighting the value of regional studies.^7^ This narrative review addresses the challenges of treatment and opportunities for understanding longitudinal patterns of pain and pain transitions related to RP in the United States. The rationale for this review is that the pain component of RP is not included in current treatment guidelines that focus on vasodilation, and advances in outcome measures can inform a state‐of‐the‐art approach to studying this symptom.

**SIGNIFICANCE & INNOVATIONS**

- Recent significant advances in the development of patient‐reported outcomes, which are grounded in the patient experience, allow for proper quantification of Raynaud phenomenon (RP) in systemic sclerosis and can allow for understanding of longitudinal patterns of pain and pain transitions related to RP.
- The use of vasodilators for pain management in the United States is significantly impacted by access to off‐label therapies not approved by the US Food and Drug Administration for this indication.
- There are innovative new assessment tools, such as automated interpretation of capillaroscopy reports and remote training, that may assist in improved diagnosis.
- In this review article, new mechanistic insights for pain management with vasodilator treatment of RP are discussed.

## Methods

A comprehensive search of published RP treatment guidelines up until September 1, 2025, was performed. Search databases included Medline (PubMed), Embase, and Scopus for the index terms “Raynaud's phenomenon treatment guidelines” OR (Raynauds AND phenomenon AND treat/exp OR treatment) AND (guidelines/exp OR guidelines). Articles that were diagnostic guidelines, treatment systematic reviews or meta‐analyses of randomized trials, poster presentations, and non‐English guidelines were excluded. If a society guideline was updated, only the most recent was included. Eligibility, data extraction, and quality assessment were subject to review by two independent reviewers (TMF and CGF), with a third reviewer resolving discrepancies (JB).

## Results

**Figure 1:**
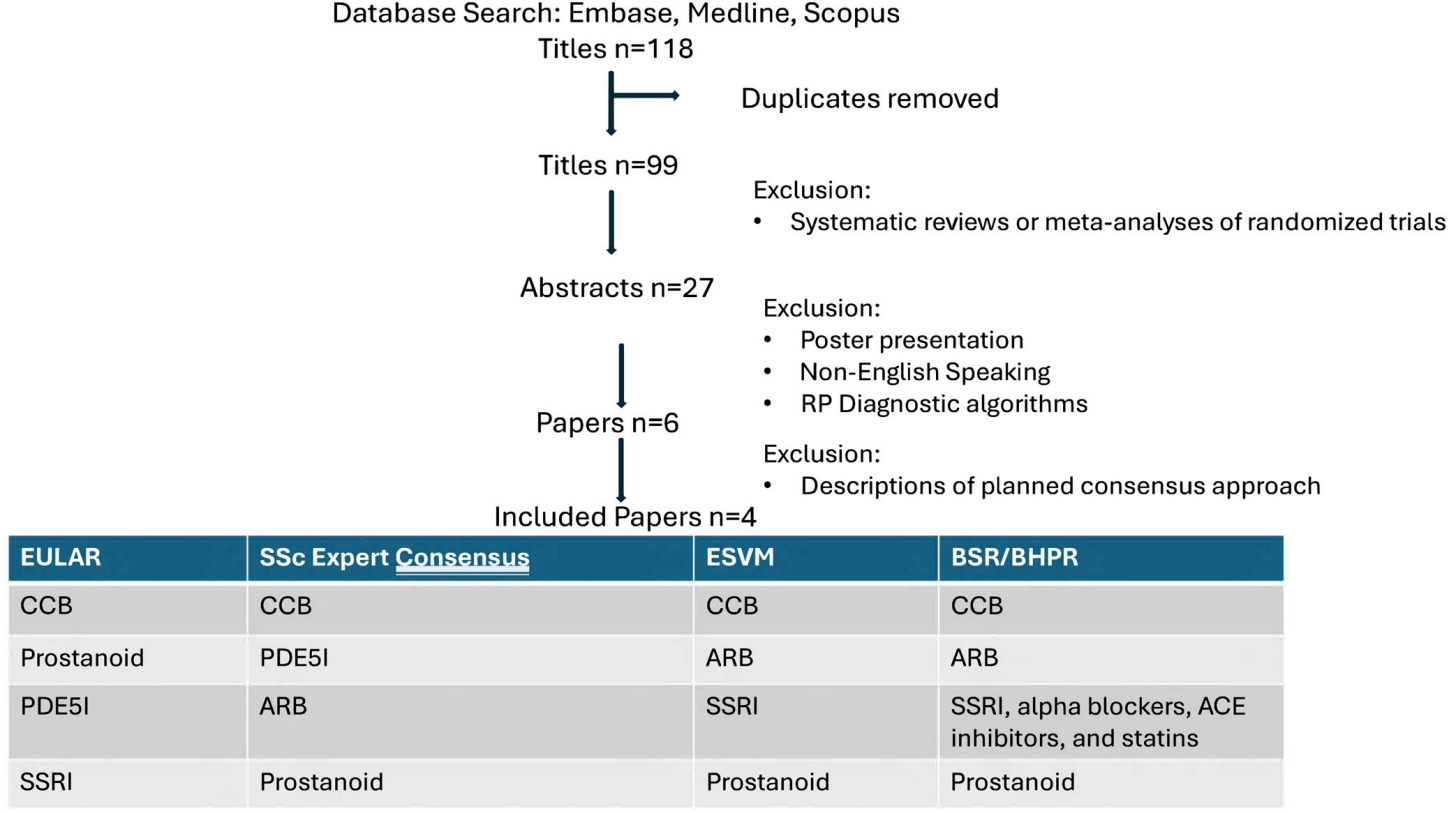
Narrative review of published treatment guidelines for RP. ACE, acetylcholine esterase; ARB, angiotensin receptor blocker; BSR/BHPR, British Society for Rheumatology/British Health Professionals in Rheumatology; CCB, calcium channel blocker; ESVM, European Society for Vascular Medicine; PDE5I, phosphodiesterase 5 inhibitor; RP, Raynaud phenomenon; SSc, systemic sclerosis; SSRI, selective serotonin reuptake inhibitor. Color figure can be viewed in the online issue, which is available at http://onlinelibrary.wiley.com/doi/10.1002/acr.25660/abstract.

**Figure 2:**
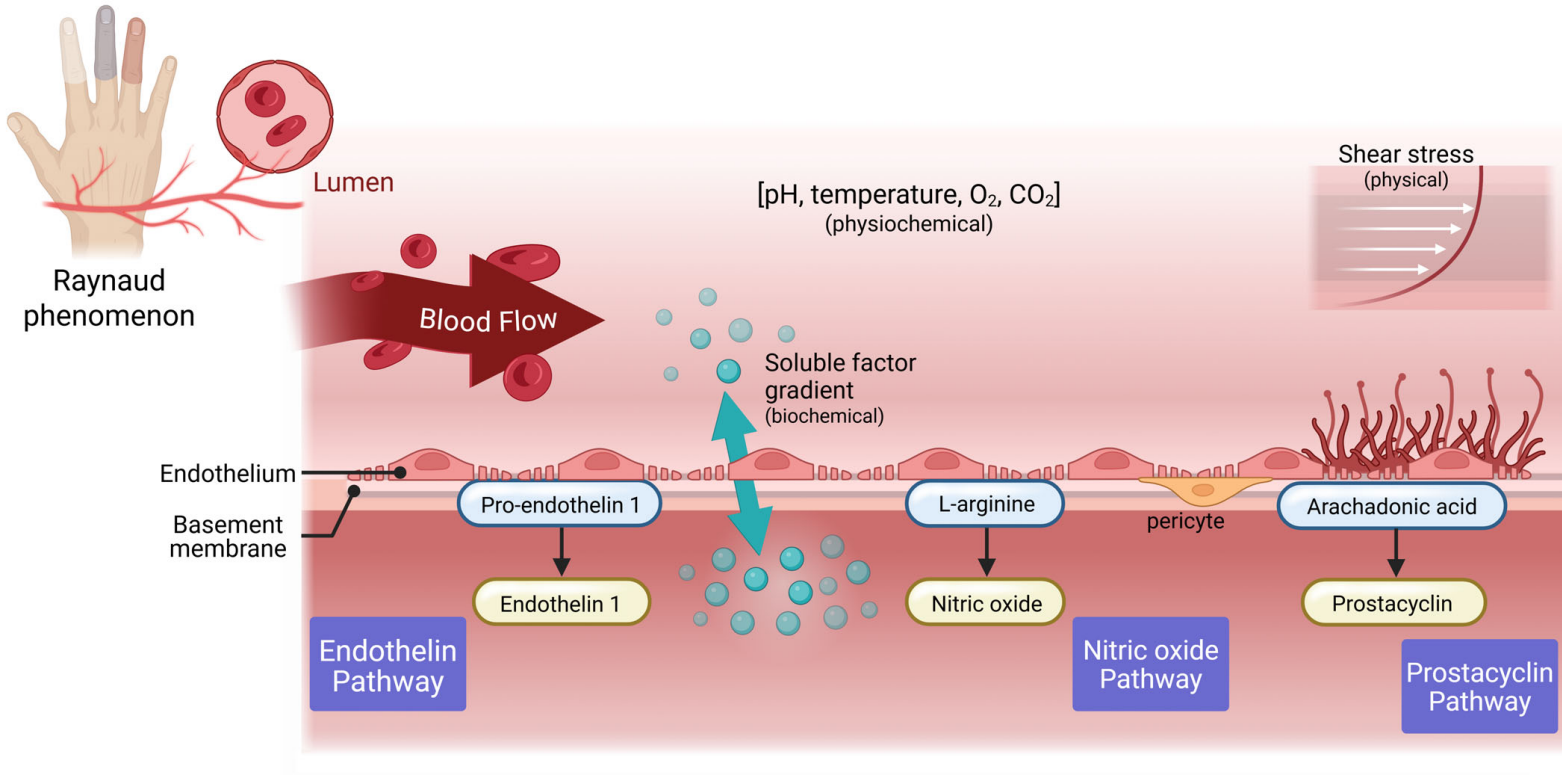
Mechanism of action should address the role of barrier function of vascular endothelium with connective tissue.

As shown in Figure 1, 118 published articles were identified by the search terms “Raynaud's phenomenon treatment guidelines,” and 27 abstracts were reviewed. One potentially eligible abstract from the Chinese Rheumatology Association was excluded as non‐English^8^; others were diagnostic (not treatment) recommendations or a poster. Four articles were published as RP treatment recommendations or guidelines. These articles included the EULAR 2023 recommendations for the treatment of SSc,^9^ the European Society for Vascular Medicine 2017 recommendations for the diagnosis and management of RP,^2^ 2018 treatment algorithms by consensus of SSc experts,^10^ and the British Society for Rheumatology/British Health Professionals in Rheumatology 2016 guideline for treatment of SSc^11^ (Figure 2). Pain management for RP is not included in any of the guideline‐based recommendations. Intravenous prostanoid therapy for RP is in all published guidelines but is cost‐prohibitive in the United States for routine use.

## What is already known about this subject?

There are guidelines for the treatment of RP^2^, ^9^, ^10^, ^11^ designed to escalate therapies for severity. The guidelines focus on CTD‐related RP and include use of prostanoids, which is not covered by insurance companies in the United States and is thus cost‐prohibitive. Although patient education and lifestyle adaptations are first‐line, guideline‐based treatments for RP, access to the wide range of pharmacological options outlined in international guideline‐based care for RP can be difficult in the United States because there are no US Food and Drug Administration (FDA)–approved drugs for this indication. Although treatment of RP aims to minimize the occurrence of painful episodes and prevent hand‐related disability, the medication management in guidelines focuses on vasodilators. It is also noted that some medications are associated with exacerbation of RP and thus should be stopped if digital symptoms worsen.^12^, ^13^ These medications can include stimulants taken for attention deficit disorder, certain migraine medications, and beta‐blockers. A medication review is a critical aspect of clinical trial exclusion criteria for RP.

The FDA requires pharmaceutical intervention to achieve significance in impacting feel, function, or survival for approval. The regulatory authorities in the United Kingdom (Medicines and Healthcare products Regulatory Agency), the European Union (European Medicines Agency), and Japan (Pharmaceuticals and Medical Devices Agency) have approved the use of calcium channel blockers (CCBs), phosphodiesterase 5 inhibitors (PDE5‐I), endothelial receptor antagonists, and prostacyclin analogues for the treatment of RP related to SSc. Since1980 there have been several trials designed to test treatment of RP and its severe manifestation of digital ulceration (DU). Key aspects of previous studies include identification that a clinical trial length should be specific to cold‐weather months to capture drug effect, global representation of study participants but regional subanalysis to capture environmental effects, and stratification of RP severity, including failed prior therapies and disease duration of SSc. A critical component of outcome measures that are used in clinical trials is the ability to quantify disease activity versus prevention or occurrence of irreversible damage.

Nailfold capillaroscopy is used diagnostically to determine if RP is related to CTD. The importance of this diagnostic test highlights that RP secondary to CTD can result in tissue damage, such as DU. An abnormal capillaroscopy result in the setting of SSc‐specific antibodies has a high probability of the patient meeting classification criteria in the upcoming five years. Thus, this screening procedure is critical for rheumatologists to perform. However, there are no formal US‐based training programs. Historically, this is because capillaroscopy is not a billable procedure to insurance companies, and the gold standard equipment to perform capillaroscopy is expensive and time consuming to use in clinical practice. Advances in low‐cost image acquisition with automated interpretation, as well as online training programs, have made this critical diagnostic tool feasible. Nailfold capillaroscopy also may be helpful for RP clinical trial enrichment, as capillary density dropout is strongly associated with DU, which is a painful end‐stage manifestation. The correlation of other nailfold abnormalities, such as the presence of microhemorrhage and abnormal size and shape of the vessels, with pain is less clear. Nonetheless, with the reduction of costs of devices that capture nailfold images, online training offered by the EULAR Study Group on Microcirculation in Rheumatic Diseases, and decentralized capillaroscopy interpretation offered by cloud‐based systems,^14^, ^15^ US‐based rheumatologists will be able to use nailfold capillaroscopy reliably. The lack of nailfold videocapillaroscopy as a diagnostic and prognostic tool in clinical practice has potentially impeded US‐based RP studies.

## What does this study add?

There are advances in outcome measures that capture pain, which can be used in RP clinical trials. The concept of how a therapeutic intervention impacts how a patient feels is a critical aspect of FDA regulatory approval and underscores the importance of understanding RP‐related pain. Attack rates, severity scores, participant‐preference scores, and physiologic measurements have underperformed in randomized control trials (RCTs).^16^, ^17^ Furthermore, the presence of pain related to RP may impact a patient's willingness to take therapeutics or enroll in a clinical trial with this symptom or a resultant DU as the primary outcome, adding selection bias to possible RCT design. As such, easy‐to‐use instruments to measure RP must be sensitive to change, reliable, valid, and grounded in the patient experience.^18^ The lack of patient involvement in the conceptual framework, domain generation, item generation, cognitive interviewing, and respondent burden has been the main criticism of the Raynaud's Condition Score (RCS), which is the most widely used instrument in clinical trials studying moderate to severe RP.^19^ The RCS uses a 0 to 10 Likert scale for patients to document in a daily diary their assessment of the combination of severity, frequency, duration, and impact of their RP for that day.^20^ The reporting value is the mean of two weeks of daily ratings; thus, as a composite assessment, the RCS does not adequately capture the potential shorter‐term impact of drug effect on symptoms, which has critical importance of assessment of “feel” for FDA drug approval.^21^, ^22^ When the RCS diary threshold was applied to patients with SSc in a real‐world population, it failed to meet all three criteria of feel, function, and quality of life, highlighting the inability to use this outcome measure to enrich RP clinical trials for regulatory approval.^23^, ^24^ An additional concern is that RP outcome measures that incorporate a patient‐perceived functional status can be impacted by coping strategies independent of prescribed therapeutics, which highlights that a focus on measures that capture impact on pain is a critical aspect of RP severity assessments.^25^, ^26^ Furthermore, RP clinical management and RP intervention trial designs should consider temperature patterns.^27^

RP outcome measures that capture how patients feel and function might prove superior to diary‐based approaches for assessing condition severity. An international multicenter validation study of the 27‐item Assessment of Systemic Sclerosis‐Associated Raynaud's Phenomenon (ASRAP) and the 10‐item short form (ASRAP‐SF) showed the questionnaires are valid and reliable novel patient‐reported outcome measures for assessing the severity and impact of SSc‐RP.^28^, ^29^ The ASRAP questionnaires had good correlation with instruments for assessing disability, hand function, global health assessment, and perhaps most importantly, if used for general RP assessments, intensity of pain. The benefit of this instrument is that it was developed with patient partners with SSc. Its use in other CTD‐related RP or primary RP is not proven. As such, pain numerical rating scales or diaries may provide better measurement if used in all RP subsets regardless of etiology.

Although patient reports can capture RP symptom characteristics, objective assessment of digital microvascular function and morphology is helpful for understanding potential mechanisms of disease. Measurement of the temperature of the digits and thermal gradient (temperature of digits minus temperature of dorsum of the hand) may help discern treatment effect in RP.^30^ In conjunction with nailfold capillaroscopy, point‐of‐care thermographic imaging is a noninvasive quantitative tool for assessing peripheral vasculopathy.^31^ Functional microcirculatory changes are evident in both primary RP and RP‐related to CTD, thus an important aspect of assessment; however, it is noted that pain and paresthesia are more common in RP related to CTD.^32^, ^33^ This distinction of painful blanching of the skin guiding prescribed therapies is important contextually for understanding guideline‐based care for RP treatment.^2^, ^9^, ^34^

## How might this impact clinical practice or future developments?

Including pain‐related mechanisms for RP allows expansion of studies on the mechanism of action of therapies prescribed for RP. RP is considered a disorder of vascular thermoregulatory control. In non–CTD‐related RP, in which nailfold capillaroscopy is normal, vasospasm of the digital and cutaneous vessels is believed to occur because of an increased α_2C_‐adrenergic response located on vascular prejunctional terminals and smooth muscle and does not result in vascular pathology.^35^ As such, a drug targeting vascular thermoregulatory control mechanisms is important for RP symptomatic modification. In CTD‐related RP, in which vascular structural and functional changes, combination therapies which address symptoms and vascular damage may be needed.

Preclinical models of CTD‐related RP and SSc delineate the relative contributions of specific ligands, receptors, their signaling pathways, and feedback mechanisms and have been challenged by lack of fidelity for features of vasculopathy, fibrosis, and autoimmunity. Skin biopsies support the importance of abnormal endothelium and microvascular pericytes in CTD‐related RP, which are not present in primary RP. Unbiased spatial proteomics with single‐cell resolution in skin biopsy tissues further support the role of the endothelial cell in patients with SSc that expresses markers for endothelial‐to‐mesenchymal transition and is located in close proximity to immune cells and myofibroblasts.^36^ Specifically, pericytes are perivascular mesenchymal stem cells with macrophage‐like properties embedded within the endothelial basement membrane and are found primarily around blood capillaries, precapillary arterioles, postcapillary venules, and collecting venules to facilitate and assimilate cell communication. The neurotransmitter noradrenaline, released by sympathetic nerve endings, activated α_2_‐adrenoceptor on pericytes, which leads to vessel constriction.^37^ The potential of vascular therapeutics that can target the pericyte is intriguing. It is critically important to understand the role of immune‐mediated vascular dysregulation in CTD‐related RP pain.

Therapeutics for RP can target the vascular lumen, barrier function, smooth muscle, or surrounding connective tissue (Figure 2). The wall‐to‐lumen ratio of the radial artery in primary RP and SSc‐related RP is reduced and functionally impaired.^38^, ^39^ Treatment with an essential cofactor for endothelial nitric oxide (NO) can improve endothelial function in SSc^40^, ^41^; however, accumulating evidence supports that the interplay between carbon monoxide, generated by heme oxygenase and NO plays a crucial role in vascular homeostasis and regeneration by improving endothelial cell communication with neighboring cells, including smooth muscle cells, immune cells, and pericytes.^42^ Nifedipine, a first‐line therapy and one of the most widely used pure L‐type CCBs for treatment of patients with RP, is hypothesized to reduce osteoblastic differentiation of pericytes, thus reducing vascular calcification that may be important for calcinosis. Of note, cilnidipine is a newer fourth‐generation CCB with both L‐type and N‐type Ca^2+^ channel blockade that has renoprotective, cardioprotective, and neuroprotective effects through direct effect on sympathetic nerve endings.^43^ Cilnidipine has potentially clinically relevant activity at Nav1.7, a target in pain treatment, and is effective as a combination therapy with PDE5‐I.^44^ Cilnidipine is currently under investigation for SSc‐RP efficacy, safety, and SSc‐RP–related pain.

In severe RP or failing first‐line CCB treatment, prescription of a PDE5‐I, which protects cGMP from degradation‐mediating vascular smooth muscle relaxation through NO signaling, is considered second‐line therapy for RP.^45^ Of interest, the mechanism of action of pentoxifylline, which can be consider in RP, may be related to phosphodiesterase inhibition in addition to platelet sensitivity to the antiaggregatory action of endogenous prostaglandin I_2_, which is an unstable cyclooxygenase metabolite detected first in vascular endothelial cells and is associated with the amelioration of pericyte reduction and the transition to myofibroblasts.^46^ Although PDE4‐I has not been specifically studied in RP, the PDE4 subfamily (PDE4A, PDE4B, PDE4C, and PDE4D) selectively degrades cAMP and plays a vital role in regulating the balance of second messengers in various tissues.^47^ PDE4 is the major subtype of PDE enzymes expressed in immune and inflammatory cells and is under investigation in SSc interstitial lung disease for the potential effect of reversibility of vascular‐mediated fibrosis.^48^

Prostacyclin analogues, most commonly intravenous iloprost, are reserved for severe RP, usually after CCB and PDE5‐I treatments fail, but have cost‐prohibitive prescription restrictions in the United States when used for the primary indication of RP.^45^ Fluoxetine, a serotonin reuptake inhibitor, is a cost‐effective alternative treatment for mild RP when blood pressure side effects limit vasodilators. Experimental data suggest that serotonin drives fibrosis in the skin and visceral organs, promotes platelet aggregation, induces vasoconstriction, and increases pulmonary vascular resistance; thus, it remains an interesting therapeutic target for CTD‐related RP.^49^, ^50^ Local therapies such as digital Botox injections may prevent the oxidant‐induced intracellular accumulation of reactive oxygen species in vascular endothelial cells and may be considered an important pain management strategy.^51^, ^52^ The identification of neuropathic pain may benefit from adjuvants such as gabapentinoids and antidepressants, with temporary use of opioids sometimes required in severe cases with digital ischemia.^53^

## Opportunities for clinical trials for RP

It is critical to study primary and CTD‐related RP concurrently to clarify medication effectiveness, but it is important to stratify patients based on capillaroscopy, disease severity, and duration. The FDA defines a basket trial as a master protocol study designed to test a single investigational drug or drug combination in different populations defined by disease stage, histology, number of prior therapies, genetic or other biomarkers, or demographic characteristics. This design allows a strong response signal seen in a substudy (ie, SSc‐RP) to allow for expansion to generate further data to support regulatory approval.^54^ The use of platform clinical trial design may be possible in unique populations, such as the Veterans Health Administration (VHA). Our previous study data from the VHA suggests that CCB medications are potentially being underused for RP and SSc treatment.^55^ Furthermore, well‐defined disease populations, such those in the Collaborative National Quality and Efficacy Registry for SSc, allow integration of disease duration into outcome assessments, such as pain related to RP and DU.^56^

## Conclusions

Treatment of pain related to RP is a challenge for health care providers in the United States due to diagnostic and training limitations and therapeutic access to guideline‐based vasodilator medications, such as cost‐prohibitive prostanoids. The use of capillaroscopy can allow the identification of patients with CTD who require medication therapy. Advances in outcome measures, such as the ASRAP, will allow improved clinical trial design for RP that can meet FDA rigor for significant impact on feel and function. Preclinical models and secondary outcomes, which focus on pericyte biology and mechanism of fibrotic reversal, offer hope to patients with SSc with painful DU. Clinical trial design should include both primary and CTD‐related RP to clarify therapeutic effect and the mechanism of painful skin blanching to best understand patterns of pain‐related to RP in the United States.

## AUTHOR CONTRIBUTIONS

All authors contributed to at least one of the following manuscript preparation roles: conceptualization AND/OR methodology, software, investigation, formal analysis, data curation, visualization, and validation AND drafting or reviewing/editing the final draft. As corresponding author, Dr T. M. Frech confirms that all authors have provided the final approval of the version to be published and takes responsibility for the affirmations regarding article submission (eg, not under consideration by another journal), the integrity of the data presented, and the statements regarding compliance with institutional review board/Declaration of Helsinki requirements.

## Supporting information


**Disclosure form**.
